# Lentinan progress in inflammatory diseases and tumor diseases

**DOI:** 10.1186/s40001-023-01585-7

**Published:** 2024-01-03

**Authors:** Guangda Zhou, Haiyan Liu, Ying Yuan, Qian Wang, Lanping Wang, Jianghua Wu

**Affiliations:** 1 Neck-Shoulder and Lumbocrural Pain Hospital of Shandong First Medical University, Jinan, 250062 China; 2 Department of Ultrasound, The Affiliated Taian City Central Hospital of Qingdao University, Taian, 271000 China; 3 Department of Neurology, Xingtai Third Hospital, Xingtai, 054000 China; 4 Department of Central Laboratory, The Affiliated Taian City Central Hospital of Qingdao University, Taian, 271000 China; 5 Department of Surgery, The Second Affiliated Hospital of Shandong First Medical University, Taian, 271000 China; 6 School of Nursing, Shandong First Medical University & Shandong Academy of Medical Sciences, Taian, 271000 China

**Keywords:** Lentinan, Inflammatory diseases, Tumor, Cancer

## Abstract

Shiitake mushrooms are a fungal food that has been recorded in Chinese medicine to nourish the blood and qi. Lentinan (lLNT) is an active substance extracted from shiitake mushrooms with powerful antioxidant, anti-inflammatory, anti-tumor functions. Inflammatory diseases and cancers are the leading causes of death worldwide, posing a serious threat to human life and health and posing enormous challenges to global health systems. There is still a lack of effective treatments for inflammatory diseases and cancer. LNT has been approved as an adjunct to chemotherapy in China and Japan. Studies have shown that LNT plays an important role in the treatment of inflammatory diseases as well as oncological diseases. Moreover, clinical experiments have confirmed that LNT combined with chemotherapy drugs has a significant effect in improving the prognosis of patients, enhancing their immune function and reducing the side effects of chemotherapy in lung cancer, colorectal cancer and gastric cancer. However, the relevant mechanism of action of the LNT signaling pathway in inflammatory diseases and cancer. Therefore, this article reviews the mechanism and clinical research of LNT in inflammatory diseases and tumor diseases in recent years.

## Introduction

Shiitake mushrooms are nutrient-rich, widely cultivated fungal plants, and their active extracts are also widely used as traditional Chinese medicine in the treatment of various diseases [1]. A large number of pharmacological and clinical studies have shown that lentinan (LNT) that one of the active shiitake mushroom extracts, has antioxidant, immunomodulatory, anti-tumor, anti-cancer, hypoglycemic, hypolipidemic and other biological activities [2–5]. LNT are macromolecules with a β-1,3-D-glucan and its unique molecular structure is closely related to its pharmacological activity, and the glucan of the β-glycosidic bond is the key structure for its antitumor function [6, 7]. LNT has been approved as an adjuvant therapy for cancer in Japan and China [3, 8]. However,the in vivo pharmacokinetics of LNT and the molecular mechanism of anti-inflammatory and anti-cancer have not been explained [9].

Inflammatory diseases can be defined as a general term for a large group of diseases, including inflammation in various systems [10]. The severity of which has different effects on people. Light cases can heal themselves, and severe cases can endanger life, such as COVID-19, which has major damage to the global health system [11]. Malignant tumors pose a serious threat and harm to human life and health worldwide [12–14]. Genetics and lifestyle are the main causes of cancer [15]. In addition, there is a close link between inflammation, disease and cancer, and studies have confirmed that inflammation plays an important role in the development and development of tumors [16–18]. Recent studies have shown that lentinan has great potential in the prevention and trement of inflammatory diseases and cancer, with few side effects and good effects [19, 20]. Thus, this study will summarize the molecular mechanism of LNT in the prevention and treatment of inflammatory diseases and cancer, as well as the latest progress in combination with other therapeutic methods.

## Studies of LNT in inflammatory diseases

**Fig. 1:**
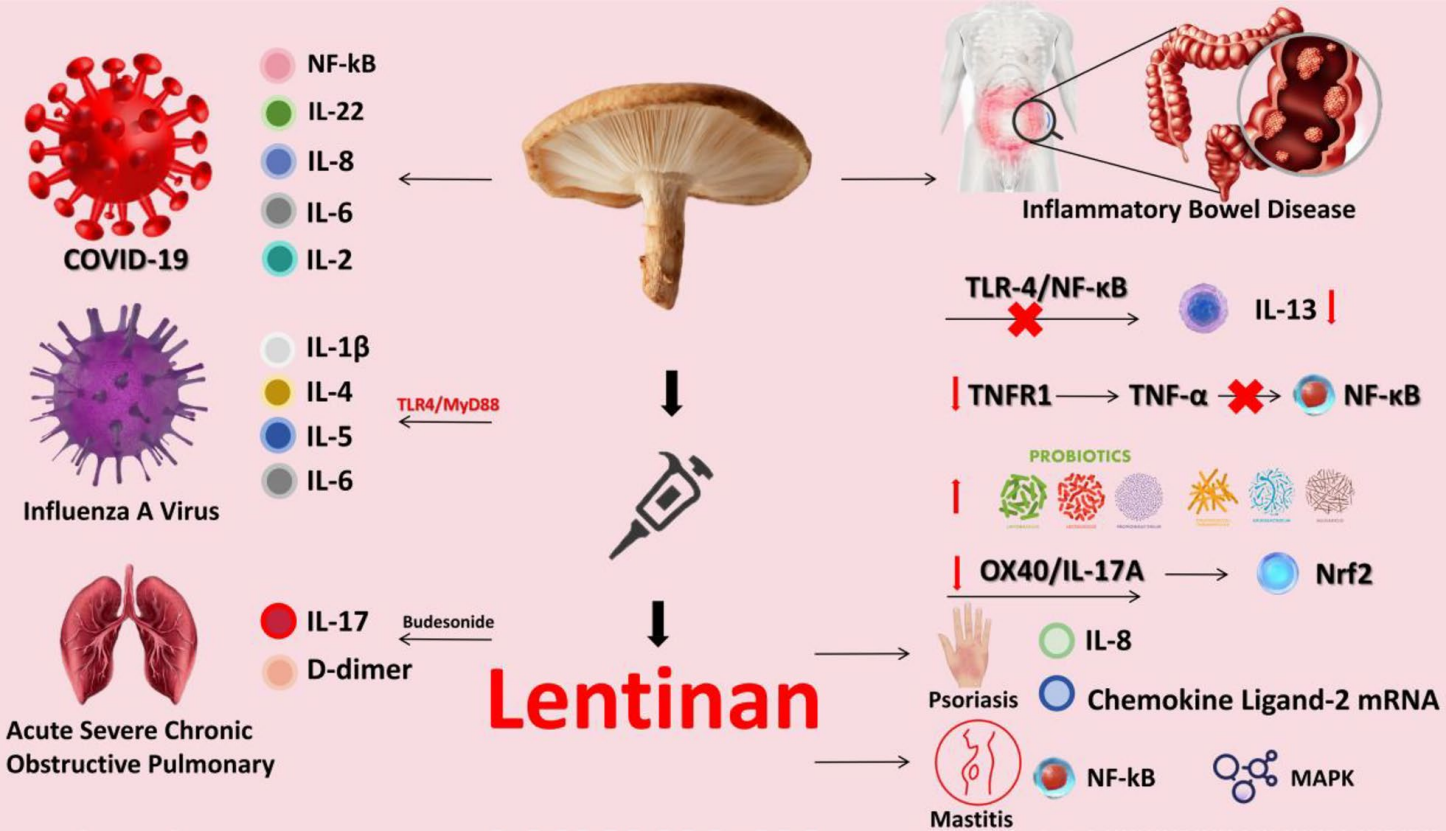
The therapeutic effects of lentinan between various types of inflammation

It is learned that LNT products have been approved for marketing as prescription drugs, including tablets, capsules, oral agents and injections, for the treatment of various diseases such as chronic viral hepatitis from China's National Medical Products Administration [21, 22], However, the clinical efficacy of LNT in the treatment of inflammatory diseases has been poorly reported. Figure 1 illustrates the therapeutic effects of lentinan between various types of inflammation.

### Studies of LNT in inflammation of intestinal inflammation.

Inflammatory bowel disease (IBD), a chronic inflammation of the gastrointestinal tract, including ulcerative colitis and Crohn's disease, has become a global disease [23]. Toll‐like receptors (TLRs) are the critical mediators of innate host defenses in the intestine that maintain mucosal immune homoeostasis. Nuclear factor kappa-B (NF- κB) is a transcription factor that regulates a variety of cell behaviors, such as pro-inflammatory responses and immune responses [24]. An experiment found that LNT can reduce the expression of pro-inflammatory cytokines such as IL-13 by inhibiting TLR-4 signaling/NF-κB signaling and high-dose LNT treatment was more effective than the positive control SASP in improving histological scores for colitis, they also found that LNT could also improve the imbalance of gut microbial colonies [25]. Another study confirmed that LNT exerts its anti-inflammatory effect by downregulating cell surface TNFR1 to inhibit TNF-α-induced NF-κB activation [26].

Dysbiosis of the gut microbiota is an important cause of IBD and a side effect of antibiotic abuse. Studies have shown that LNT can increase the number of probiotics and inhibit the NF-κB signaling pathway to improve the dysbacteriosis of intestinal microbiota caused by improper use of antibiotics [25]. In a mouse model of colitis, administration of LNT induces migration of CD4 cells from ileum to colon. Moreover,both oral and rectal administration inhibits dextran sulfate sodium (DSS)-induced colitis [27]. OX40, named CD134 or tumor necrosis factor receptor superfamily member 4 (TNFRSF4), is defined as a biomarker of T cell activation capable of increasing pro-inflammatory cytokines [28]. The latest study found that LNT can downregulate OX40/IL-17A signaling and activate nuclear factor erythroid 2 related factor (Nrf2), an important factor in oxidative stress [29].

### Studies of LNT in inflammation of other systems

Oxidative stress and cytokine storming (overexpression of pro-inflammatory cytokines) are important pathogenesis of infectious diseases, especially coronary pneumonia [30]. LNT can reduce oxidative stress-induced apoptosis, and also reduce the expression of pro-inflammatory factors such as TNF-α, IL-8, IL-2, IL-6, IL-22, thereby reducing NF-kB activation, which proves its great potential in the treatment of COVID-19 [31]. Similarly, another study found that LNT can inhibit the overexpression of cytokine storms of TNF-α, IL-1β, IL-4, IL-5, and IL-6 in influenza A viral by modulating the TLR4/MyD88 signaling pathway [32] More interestingly, a randomized controlled human experiment showed that LNT combined with budesonide reduced endogenous anti-inflammatory factor adiponectin IL-17, D-dimer acute severe chronic obstructive pulmonary in patients [33]. Moreover, an animal experiment found that LNT could inhibit oxidative stress in bovine mammary epithelial cells and also reduce the expression of inflammatory factor protein by inhibiting NF-kB and mitogen-activated protein kinase (MAPK), suggesting its great potential in the treatment of mastitis [34].

Inflammatory skin diseases are common skin diseases in life, including psoriasis which imposes a heavy psychological burden on the patients and their families and increases the risk of developing mental disorders [35]. Benzo (a) pyrene is a harmful substance that not only accelerates skin oxidation but also has a risk of causing skin cancer.A study found that Lentirosean was able to inhibit benzo (a) pyrene-induced oxidative stress in human immortalized keratinocytes (HaCaT cells) and significantly reduced IL-8 and chemokine ligand-2 mRNA [36]. Multiple sclerosis is an autoimmune disease and chronic inflammatory disease, and studies have found that LNT can regulate dectin-1 receptors, TNF-α and IL-1β to promote the transformation of M1 cells into M2 cells [37–40]. In experiments with mice with enterogenic sepsis, LNT was able to reduce the activation of TNF-α, IL-1β, IL-6 and regulate NF-κB signaling, and also reduced liver oxidative stress damage caused by enterogenic sepsis [41]. It is similar that LNT was able to reduce TNF-α, IL-1β, IL-6 in an acute kidney injury model of sepsis [42]. Extracellular regulatory protein kinases (ERK) include ERK1 and ERK2, which are involved in cell proliferation and differentiation and apoptosis. Forkhead Box Protein O1 (FOXO1), a transcription factor, is a key transcription factor involved in regulating cell proliferation, survival, DNA repair, cell cycle, apoptosis, metabolism, and immune regulation [43]. Another study found that LNT could reduce IL-10 in burned mice by regulating Erk-FoxO10 signaling [44].

## Studies of LNT in tumor-related diseases

**Fig. 2:**
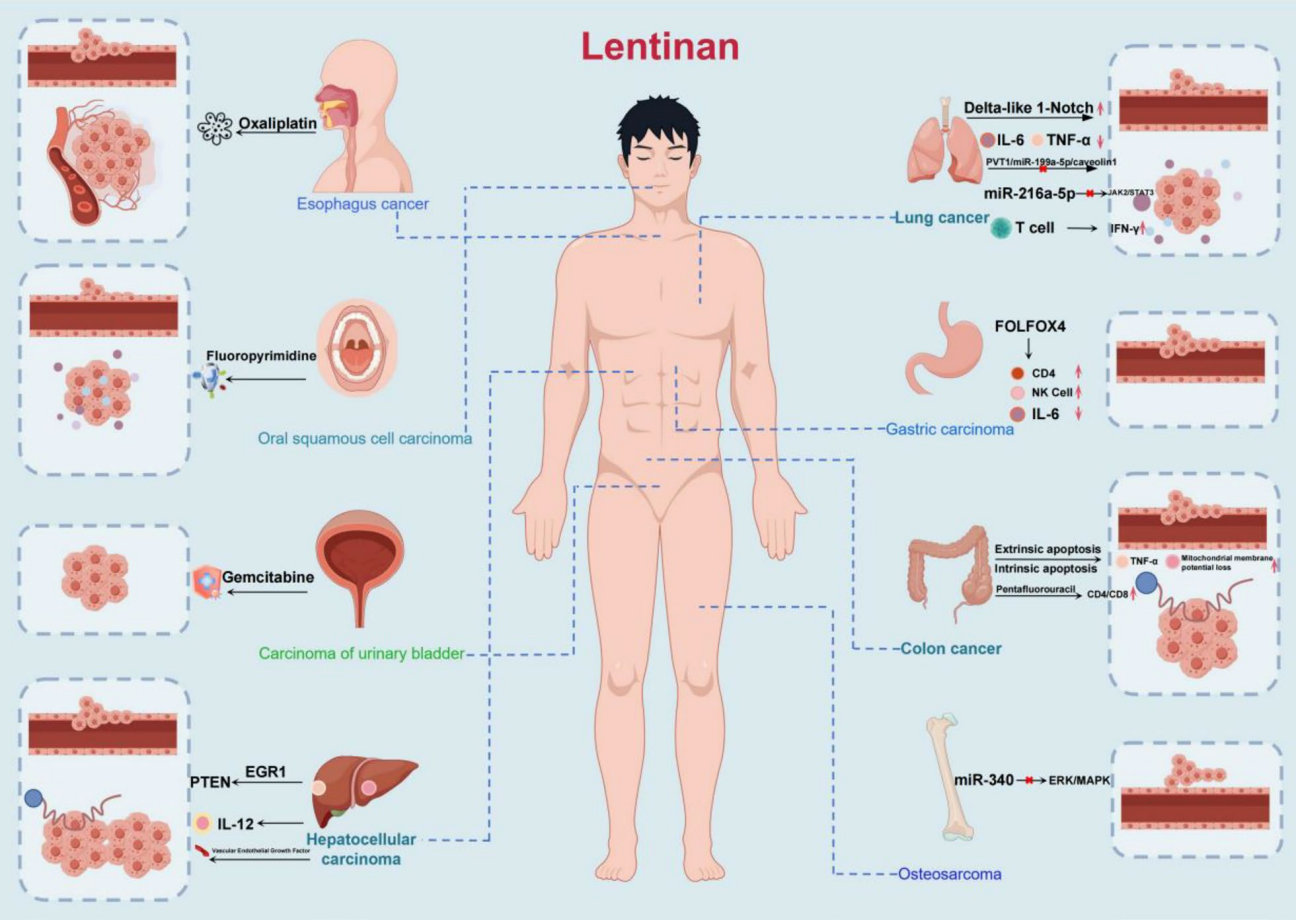
The therapeutic effects of lentinan on various tumors

As shown in Fig. 2, lentinan has therapeutic effects in various tumors.

### Studies of LNT in Lung cancer

LNT has been widely studied in cancer-related diseases such as cancer. Lung cancer is the type of cancer with high morbidity and mortality, the Lancet study shows that about 8 million people worldwide are diagnosed with lung cancer each year, and the 5-year survival rate of lung cancer is generally lower by 4%-17% [45]. Particulate matter 2.5 (PM2.5) is an air pollutant It is closely related to lung cancer, which may cause lung cancer by inducing cellular inflammatory factors and oxidative stress [46].

CD8T cells are an important member of the T lymphocyte family that can mediate specific immunity for specific killing of target cells. Xu et al. found that LNT could enhance the activation of selective activation of Delta-like 1-Notch signaling and enhance its effect on lung tumor suppression [47]. Caveolin-1, one f the components of caveolae play an important role in a variety of cellular functions such as tumorigenesis cell signaling and it is regulated by miR-199 [48]. miR-199a-5p is considered a tumor suppressor, and plays an important role in the development of lung cancer. As microRNA sponges, long noncoding RNA plasmacytoma variant translocation 1 (PVT1) also plays an important role in lung cancer development [49]. Further, PVT1 promoted caveolin1 expression by absorbing miR-199a. LNT can inhibit PM2.5-induced expression of IL-6 and TNF-α as well as the PVT1/miR-199a-5p/caveolin1 Pathway in Lung Cancer [50]. Janus kinase2/signal transducer and activator of the transcription3 (JAK2/STAT3) signaling pathway plays an important role in the growth, migration and transformation of cancer cells, and studies have shown that LNT can inhibit JAK2/STAT3 signaling pathway by regulating miR-216a-5p, thereby promoting apoptosis in lung cancer [51]. Tumor angiogenesis is an important condition for tumor growth and metastasis, therefore, anti-angiogenesis is gaining more and more attention in tumor treatment [52]. A research has found that LNT can enhance T cell immune function and promote the expression of anti-vascular factor interferon γ (IFN-γ), and the anti-tumor effect of low-dose LNT is more significant than that of high-dose LNT [53].

In addition, the clinical effect of LNT in treating lung cancer has been widely reported. It is known that cellular immunity mainly relies on T lymphocytes, and the ratio of suppressor T cells and helper T cells determines the level of immune function, and if the CD4/CD8 ratio is elevated, tumor growth will be inhibited. Zhu et al. randomized controlled studies found that LNT combined with GP (guitar cesibin combined with cisplatin) chemotherapy improved the effective CD4/CD8 ratio and better quality of life in patients with advanced lung cancer compared with GP chemotherapy alone, and had fewer adverse reactions such as gastrointestinal reactions [54] Moreover, another study also found that LNT combined with GP enhanced the immune function of CD4 and NK cells in patients with advanced non-small cell lung cancer [55]. The above cell and animal experiments and clinical effects fully prove the importance of LNT in the treatment of lung cancer.

### Studies of LNT in Colorectal cancer

Colon cancer is a malignant tumor of the digestive system with high clinical incidence, surgery is currently the main treatment for colon cancer, chemotherapy and targeted therapy and other adjuvant methods are also very important, but regardless of chemotherapy and targeted therapy or combination regimens such as oxaliplatin plus capecitabine (CapeOX) There are serious adverse effects, such as bone marrow suppression, cytotoxicity, which lead to some colon cancer patients can not tolerate [56, 57]. Therefore, it is necessary to develop new drugs to treat colon cancer. In addition, autophagy is also an important pathway by which ER stress can initiate apoptosis [58]. Zhang et al. [59] showed that endoplasmic reticulum stress can exert anti-tumor effects through autophagy and apoptosis in colon cancer. The two major pathways of apoptosis are extrinsic signaling pathways, involving tumor necrosis factor receptor gene superfamily members, including FasRL/FasR, TNF-α/TNFR1, and mitochondria-induced intrinsic signaling pathways [60]. LNT can simultaneously initiate cell extrinsic apoptosis and intrinsic apoptosis in colon cance by increasing TNF-α and mitochondrial membrane potential loss [61]. Mao et al. [62] used LNT combined with fruit acid in colorectal cancer mice and found that the combination of the two could increase the expression of antitumor factors TNF-α and promote the conversion of M2 cells into M1 cells.

More importantly, the existing clinical studies have revealed the application effect of LNT in colorectal cancer.Chen et al. randomized controlled experiments found that LNT combined with chemotherapy regimen can greatly improve the effective rate (chemotherapy regimen with 28% effective rate, LNT combined with effective rate 52%) in colorectal cancer [63]. Moreover, Tu et al. also found that LNT combined with pentafluorouracil can not only enhance the immune function of colorectal cancer patients (CD4/CD8 ratio is elevated), but also significantly improve the quality of life of patients [64].

### Studies of LNT in Liver cancer

At present, significant progress has been made in the diagnosis and treatment of liver cancer, but due to clinical complexity, liver cancer still lacks effective treatment measures and has a poor prognosis [65, 66]. Sorafenib is a multi-target kinase inhibitor and a guideline-recommended first-line drug for liver cancer, which can improve the survival rate of patients, but there is drug resistance and toxicity, so clinical application is limited [67].

The EGR1 gene is an important transcription factor and belongs to the EGR protein family, which has been reported to be transcription factors in phosphatase and tensin homolog (PTEN) tumor suppressor genes, and Bcl-2 and Bax are important members of the Bcl-2 gene family and are closely related to apoptosis. Bcl-2 is considered anti-apoptotic, while Bax is a pro-apoptotic factor in the Bcl-2 family. PTEN can inhibit cancer cells by inhibiting AKT phosphorylation to promote increased expression of pro-apoptotic factors and reducing the apoptotic pathway initiated by anti-apoptotic factors. Studies have shown that LNT can activate PTEN-induced apoptosis pathways by promoting the expression of EGR1 [68].The Apoptotic protease activating factor 1 (Apaf-1) protein is an important factor in mitochondrial mediation of the cellular intrinsic apoptosis pathway [69]. Studies have found that LNT combined with cisplatin can not only reduce the dose of cisplatin, but also promote the activation of the intrinsic apoptosis pathway through the regulation of signals, leading to apoptosis of liver cancer cells [70]. Similarly, another study found that LNT in combination with oxaliplatin chemotherapy could also exert anti-tumor effects by activating the intrinsic apoptosis pathway, and also reduced the side effects of oxaliplatin chemotherapy [71].

What is more surprising is that LNT can act as antigen, activate hepatocyte immune function and anti-tumor activity, which is of great significance in liver cancer prevention [72]. However, a previous randomized controlled study showed no statistically significant difference in the survival time and mortality of patients with liver cancer compared with pentafluorouracil alone compared with control groups [73], and recent studies showed that LNT-linked hepatic artery embolization chemotherapy can significantly improve liver function enhancement IL-12 expression in liver cancer patients compared with hepatic artery embolization chemotherapy alone [74]. In addition, another randomized controlled study found that the use of LNT not only reduced the expression of vascular endothelial growth factor, but also reduced the incidence of adverse effects of chemotherapy, such as nausea, vomiting, pain, etc. [75].

### Studies of LNT in gastric cancer

Gastric cancer is a malignant tumor originating from gastric mucosal epithelial cells. Early gastric cancer is mainly treated by surgery, and advanced stage [76]. Gastric cancer chemotherapy, immunotherapy, targeted therapy, etc. have also made breakthroughs in gastric cancer. However, the common chemotherapy regimen oxaliplatin combined with pentafluorouracil has adverse reactions, and the economic cost is large, and the limited clinical application of new adjuvant chemotherapy drugs, such as anti-angiogenic drugs, has not been fully verified [76, 77].

The clinical effect of LNT in gastric cancer has been widely reported. A randomized controlled study in Japan showed that LNT combined with pentafluorouracil improved survival time for advanced gastric cancer, which is consistent with the results of a meta-study of five randomized controlled trials [78, 79]. Another prospective study also found that LNT combined with chemotherapy not only improved the survival time of patients with advanced unresectable gastric cancer (199 days in the control group and 277 days in the shiitake combination chemotherapy group) but also improved the quality of life of patients [80]. A study from China showed that LNT combined with chemotherapy FOLFOX4 can improve the treatment efficiency of elderly patients with advanced gastric cancer, and significantly enhance the immune function of CD4 cells, increase NK cells and reduce IL-6 levels [81]. Furthermore, another study showed that LNT combined with paclitaxel chemotherapy could not only improve the efficiency of advanced gastric cancer, but also reduce the toxic effects of chemotherapy such as leukocytopenia, gastrointestinal reactions, etc.) [82]. Although LNT has been approved in Japan as an immune agent for chemotherapy in gastric cancer, the signaling pathway and mechanism of LPS in the treatment of gastric cancer need further research [83].

### Studies of LNT in other cancer

Studies have shown that LNT can inhibit the ERK/MAPK signaling pathway by regulating miR-340, thereby promoting apoptosis in osteosarcoma cells [84]. Esophageal cancer is important in cancer-related deaths in men. At present, surgery and chemotherapy are still the main treatment methods, and there is currently no effective treatment for advanced esophageal cancer [85]. Studies have shown that LNT combined with oxaliplatin can effectively promote apoptosis of esophageal cancer cells [86]. It is gratifying that a randomized controlled experiment shows that LNT combined with tigafluoride not only reduced the level of pro-inflammatory factors in esophageal cancer patients, but also improved symptoms and quality of life [87].

Bladder cancer is the second malignant tumor of the urinary system, and studies have shown that the incidence of bladder cancer in men is significantly higher than that in women (about 3–4 times that of women), but women are often diagnosed with advanced stages and poor prognosis [88]. A cell experiment showed that LNT combined with gemcitabine inhibited urothelial bladder cancer cells and enhanced the cytotoxic effects of gemcitabine [89]. Similarly, another in vivo and in vitro animal experiment showed that LNT in combination with fluoropyrimidine, another first-line chemotherapy drug, significantly inhibited the growth of human oral squamous cell carcinoma [90]. The above preclinical animal experiments have shown that the use of LNT alone or combined chemotherapy can significantly inhibit tumor growth in treating cancer treatment, but more clinical experiments are needed to verify its role in human cancer.

## Discussion

LNT is an shiitake extract with anti-inflammatory, antioxidant, anti-tumor and other biological activities and functions. Inflammatory diseases and cancers pose a serious threat to human health, and the current treatment methods for inflammatory diseases and cancers still cannot meet the treatment needs of patients. This article reviews the molecular mechanisms and preclinical and clinical studies of LNT in inflammatory diseases and cancer, to provide evidence for the clinical development and application of LNT.

Vaccines are biological agents made from bacteria and viruses, which can make the human body produce specific immunity, and then play a role in preventing and controlling diseases, such as influenza vaccines. Although medical technology advances and diversifies in treatment methods, cancer prevention is still very important, and the development and application of cancer vaccines are of great significance in reducing the global cancer burden. Studies have shown that LNT has great potential for vaccine delivery, acting as a vaccine adjuvant, enhancing vaccine immunity and mitigating vaccine response [91–93].

Targeted therapy is a research hotspot in tumor therapy. Especially with the study of tumor signaling pathways and the development of nanotechnology-based drug delivery systems for precise and accurate drug delivery in tumor areas, tumor cell targeted therapy is undoubtedly the dawn of cancer treatment. Studies have shown that LNT complexes are more specific and targeted in drug delivery and gene delivery [94]. In addition, Jia et al. developed LNT selenium nanoparticles to enhance their anti-tumor effects [95]. Furthermore,studies also found that LNT selenium nanoparticles can promote apoptosis by acting on specific signaling pathways [96, 97]. Wang et al. found that LNT combined with chemotherapy drug cisplatin has a synergistic effect. These all show the superiority of LNT in targeted therapy [98].

Although breakthroughs have been made in cancer treatment, new therapies such as targeted therapies, immunotherapy and other therapies have become very promising ways to fight cancer. However, chemotherapy for cancer is still the most widely used clinical therapy. However, chemotherapy drugs (platinum, fluorouracil, etc.) inevitably have side effects such as bone marrow suppression, nephrotoxicity, digestive tract toxicity, etc. [99, 100], and the economic cost of cancer is huge and the clinical benefits are limited. However, the current study shows that LNT combined with chemotherapy can not only enhance the immune function of patients, but also enhance the efficacy of chemotherapy and reduce the adverse reactions of chemotherapy. This suggests that LNT can be used as an adjuvant to chemotherapy.

However, it is important to emphasize that the translation of these findings from preclinical studies to clinical applications requires rigorous evaluation through human clinical trials [101–103]. It is unclear how LNT is metabolized in the human body and the differences in different structures and doses of LNT in the treatment of inflammatory diseases and cancers. Rigorous clinical trials can reveal the optimal dosage, duration of treatment, and potential combination therapies for LNT-targeted drugs. These trials provide an opportunity to study the mechanism of action of drugs, identify potential biomarkers that can predict treatment response, and optimize patient selection criteria for the most effective treatment outcomes. Clinical trials provide an opportunity to explore the potential synergistic effects of LNT-targeted drugs with existing treatment modalities, such as immunotherapy or conventional chemotherapy. or else ombination therapies have the potential to improve therapeutic efficacy by targeting multiple aspects of tumor cell apoptosis and immune response, resulting in a more comprehensive and effective anti-cancer effect.

Simplified explanation of Lentinan's mechanisms:

Lentinan acts like a “wake-up call” to the body’s defense system. When it enters the body, it prompts certain immune cells to become more active. Imagine these cells as guards that are on the lookout for harmful invaders like tumor cells or agents causing inflammation. Once alerted by Lentinan, these guards become more vigilant and effective in protecting the body.

Specifically, Lentinan stimulates cells known as macrophages and dendritic cells, which are like the body’s first responders. These cells then produce signals (known as cytokines) that rally more immune cells to join the fight against diseases. Additionally, Lentinan enhances the action of natural killer cells, which are specialized in directly attacking tumor cells.

For its anti-inflammatory effects, Lentinan works by reducing the body's overactive inflammatory response. It's like calming an overly aggressive reaction to harmful stimuli, thereby preventing damage to our own tissues.

Key gaps in current research and recommendations for future studies:

Pharmacokinetics and Pharmacodynamics in Humans: Despite extensive research on Lentinan's therapeutic effects, there is a notable gap in understanding its pharmacokinetics and pharmacodynamics in human subjects. Future research should focus on comprehensive clinical trials to explore how Lentinan is metabolized and distributed in the human body, determine optimal dosages, and assess long-term effects; Molecular Structure-Bioactivity Relationship: Another significant gap is the relationship between Lentinan's molecular structure and its bioactivity; Detailed studies in this area could lead to enhanced therapeutic efficacy and the development of targeted delivery methods. Investigating nanotechnology-based systems for Lentinan delivery could be particularly fruitful; Synergistic Effects with Current Treatments: While some studies have explored Lentinan's role in conjunction with other treatments, there is a need for more in-depth research into its synergistic effects, especially in cancer therapy. Future studies should examine how Lentinan interacts with and potentially enhances the efficacy of existing treatment modalities like chemotherapy and immunotherapy; Long-Term Impact and Side Effects: There is a lack of long-term studies on the impact of Lentinan treatment, particularly concerning potential side effects and the sustainability of its therapeutic benefits. Longitudinal studies focusing on these aspects would be valuable; Personalized Medicine Approach: Given the variability in response to Lentinan treatment observed in clinical settings, research into personalized medicine approaches could be beneficial. This includes studying genetic or biomarker profiles that might predict patient response to Lentinan; Broader Range of Diseases: Although Lentinan has been studied primarily in the context of inflammatory diseases and cancers, investigating its potential applications in other diseases could be a significant area for future research. By addressing these gaps, future research can substantially enhance our understanding of Lentinan's therapeutic potential and optimize its clinical applications.

In summary, although significant progress has been made in the prevention and treatment of LNT in inflammatory diseases and tumor-related diseases, our mechanism of action, signaling pathway and upstream and downstream cytokines of LNT still need to be further explored. There is also a need to apply the existing evidence to the clinic and further explore the clinical effects of lentinan in inflammatory diseases and cancer.

Tables 1 and 2 provide a comprehensive overview of molecular mechanisms in LNT use on inflammatory diseases and cancers, highlighting the tremendous potential of the LNT application. These advances offer a promising avenue for developing new treatments that can prevent and treat inflammatory diseases and cancer.

**Table 1:** Mechanism of lentinan in inflammatory diseases

Type of disease	Model	Mechanism	in vivo /in vitro	Author, year
IBD	Mouses models of colitis	Inhibit TLR4 pathway and NF‐κB Lower inflammatory cytokine IL-13 restore the structure of the intestinal microflora	In vivo,in vitro	Liu et al. 2017 [25]
IBD	Mouses models of colitis	Downregulate TNFR1 to inhibit TNF-α-induced NF-κB activation and IL-8	In vitro	Nishitani et al. 2013 [26]
IBD	Mouses models of colitis	Stimulate of type 1 helper T cell immune responses	In vivo	Minato et al. 2023 [27]
COVID-19	Human airway epithelial cells	Reduce oxidative stress-induced apoptosis and reduce pro-inflammatory cytokine expression	In vitro	Murphy et al. 2020 [31]
Influenza A viral	Mouses models influenza A viral	Regulate TLR4/MyD88 signaling pathway TNF-α, IL-1β, IL-4, IL-5, and IL-6	In vivo,in vitro	Cui et al. 2022 [32]
Chronic obstructive pulmonary	Human experiment	Lower adiponectin IL-17, D-dimer	In vivo	Sun et al. 2019 [33]
Mastitis	Cell models of mastitis	Inhibiting NF-kB and MAPK	In vivo	Meng et al. 2022 [34]
Skin diseases	Human immortalized keratinocytes	Reduce IL-8 and chemokine ligand-2 mRNA	In vivo	Zi et al. 2020 [36]
Multiple sclerosis	Demyelination mouses models	Regulate dectin-1 receptors, TNF-α and IL-1β to promote the transformation of M1 cells into M2 cells	In vivo,in vitro	Zhang et al. 2020 [37]
Enterogenic sepsis	Septic rats	Reduce the activation of TNF-α, IL-1β, IL-6 and regulate NF-κB signaling	In vivo,in vitro	Kuanget al, 2021 [41]
Acute kidney injury	Septic rats	Reduce TNF-α, IL-1β, IL-6	In vivo	Wang et al. 2016 [42]
Burn sepsis	Septic rats	Inhibit Erk-FoxO10 signaling,reduced IL-10	In vivo	Li et al. 2020 [44]

**Table 2:** Mechanism of lentinan in tumor diseases

Type of disease	Model	Mechanism	In vivo /in vitro	References
Lung cancer	Human lung cancer cell	Enhance the activation of selective activation of Delta-like 1-Notch signaling	In vivo	Xu et al. 2022 [47]
Lung cancer	Human lung cancer cell	Inhibited PVT1/miR-199a-5p/caveolin1 Pathway	In vivo	Qi et al. 2021 [50]
Lung cancer	Human lung cancer cell	Regulate miR-216a-5p inhibited (JAK2/STAT3) signaling pathway	In vitro, in vitro,	Chen et al. 2021 [51]
Lung cancer	Mouses models of lung cancer	Promote the expression of anti-vascular factor IFN-γ	In vivo, in vitro	Deng et al. 2018 [53]
Lung cancer	Human experiment	Enhanced the immune function of CD4 and NK cells	In vitro	Zhu et al. 2022 [54]
Lung cancer	Human experiment	Regulate CD4/CD8 ratio	In vivo, in vitro	Zhou et al. 2018 [55]
Colon cancer	Mouses models of lung cancer	Endoplasmic reticulum stress autophagy	In vivo, in vitro	Zhang et al. 2021 [59]
Colon cancer	Mouses models of lung cancer	Promote the expression of TNF-α Promote mitochondrial membrane potential loss	In vivo, in vitro	Wang et al. 2017 [61]
Colorectal cancer	Mouses models of lung cancer	Reduce the expression of antitumor factors TNF-α to promote the transformation of M1 cells into M2 cells	In vivo, in vitro	Mao et al. 2022 [62]
Colorectal cancer	Human experiment	Regulate CD4/CD8 ratio	In vivo	Chen et al. 2019 [63]
Colorectal cancer	Human experiment	Regulate CD4/CD8 ratio	In vivo	Tu et al. 2010 [64]
Liver cancer	Mouses models of liver cancer	Regulate EGR1/PTEN/AKT signaling	In vivo, in vitro	You et al. 2023 [68]
Liver cancer	Mouses models of liver cancer	Regulate Apaf1 apoptosis	In vivo,	Wang et al. 2021 [70]
Liver cancer	Mouses models of liver cancer	Activate the intrinsic apoptosis pathway Inhibition of NF-κB	In vivo, in vitro	Xu et al. 2022 [71]
Liver cancer	Mouses models of liver cancer	Activate hepatocyte immune function and anti-tumor activity,	In vivo,	Wang et al. 2015 [72]
Liver cancer	Human experiment	rRaeduce IL-12	In vivo,	Nan et al. 2018 [74]
Liver cancer	Human experiment	Reduce the expression of vascular endothelial growth factor	In vivo,	Zhao, 2015 [75]
Gastric cancer	Human experiment	Increase NK cells and reduce IL-6	In vivo,	Wang et al. 2018 [81]
Gastric cancer	Human experiment	Regulate CD4/CD8 ratio	In vivo,	Yan et al. 2008 [82]
Osteosarcoma	Mouses models	Regulate ERK/MAPK signaling pathway	in vivo,	Fan et al. 2021 [84]
Esophageal cancer	Mouses models	Promote apoptosis	In vivo,	Huo et al. 2022 [86]
Esophageal cancer	Human experiment	IL-5, IL-10, and IL-2 levels decrease	In vivo,	Wang et al. 2012 [87]
Bladder cancer	Mouses models	Promote apoptosis	In vivo,	Sun et al. 2015 [89]
Human-oral squamous cell carcinoma	Cell	Up-regulate the expression of OPRT mRNA	In vivo,in vitro	Harada et al. 2010 [90]

## Data Availability

Not applicable.

## Abbreviations

LNT: Lentinan
IBD: Inflammatory bowel disease
TLRs: Toll‐like receptors
NF-κB: Nuclear factor kappa-B
IL-13: Interleukin-13
TNFRSF4: Tumor necrosis factor receptor superfamily member 4
Nrf2: Nuclear factor erythroid 2 related factor
TNF-α: Tumor necrosis factor-α
MAPK: Mitogen-activated protein kinase
ERK: Extracellular regulatory protein kinases
FOXO1: Forkhead Box Protein O1
PVT1: Plasmacytoma variant translocation 1
IFN-γ: Interferon-γ
PTEN: Phosphatase and tensin homolog
Apaf-1: Apoptotic protease activating factor 1
